# Embolic strokes of undetermined source: a clinical consensus statement of the ESC Council on Stroke, the European Association of Cardiovascular Imaging and the European Heart Rhythm Association of the ESC

**DOI:** 10.1093/eurheartj/ehae150

**Published:** 2024-04-30

**Authors:** George Ntaios, Helmut Baumgartner, Wolfram Doehner, Erwan Donal, Thor Edvardsen, Jeff S Healey, Bernard Iung, Hooman Kamel, Scott E Kasner, Eleni Korompoki, Babak B Navi, Christian Pristipino, Luca Saba, Renate B Schnabel, Emma Svennberg, Gregory Y H Lip

**Affiliations:** Department of Internal Medicine, School of Health Sciences, University of Thessaly, Larissa University Hospital, Larissa 41132, Greece; Department of Cardiology III: Adult Congenital and Valvular Heart Disease, University Hospital Muenster, Muenster, Germany; Department of Cardiology (Campus Virchow), Center of Stroke Research Berlin, German Centre for Cardiovascular Research (DZHK) partner site Berlin, Berlin Institute of Health-Center for Regenerative Therapies, Deutsches Herzzentrum der Charité, Charité, Berlin, Germany; Service de Cardiologie et CIC-IT 1414, CHU Rennes, Rennes, France; Department of Cardiology, Faculty of Medicine, Oslo University Hospital, Rikshospitalet, University of Oslo, Oslo, Norway; Cardiology Division, McMaster University, Hamilton, Canada; Bichat Hospital, APHP and Université Paris-Cité, INSERM LVTS U1148, Paris, France; Clinical and Translational Neuroscience Unit, Feil Family Brain and Mind Research Institute, Department of Neurology, Weill Cornell Medicine, New York, NY, USA; Department of Neurology, Perelman School of Medicine, University of Pennsylvania, Philadelphia, PA, USA; Department of Clinical Therapeutics, Alexandra Hospital, Medical School, National and Kapodistrian University of Athens, Athens, Greece; Clinical and Translational Neuroscience Unit, Feil Family Brain and Mind Research Institute, Department of Neurology, Weill Cornell Medicine, New York, NY, USA; Department of Neurology, Memorial Sloan Kettering Cancer Center, New York, NY, USA; Interventional and Intensive Cardiology Unit, San Filippo Neri Hospital, ASL Roma 1, Rome, Italy; Department of Radiology, Azienda Ospedaliero Universitaria (A.O.U.), di Cagliari—Polo di Monserrato, Cagliari, Italy; Department of Cardiology, University Heart & Vascular Center Hamburg, University Medical Center Hamburg-Eppendorf, Hamburg, Germany; DZHK (German Center for Cardiovascular Research), partner site Hamburg/Kiel/Luebeck, Germany; Department of Medicine, Karolinska Institutet, Karolinska University Hospital Huddinge, Stockholm, Sweden; Liverpool Centre for Cardiovascular Science at University of Liverpool, Liverpool John Moores University, Liverpool Heart & Chest Hospital, Liverpool, UK; Danish Center for Health Services Research, Department of Clinical Medicine, Aalborg University, Aalborg, Denmark

**Keywords:** Embolic stroke of undetermined source, Aetiology, Atherosclerosis, Patent foramen ovale, Left atrial disease, Left ventricular disease, Valvular heart disease, Cancer

## Abstract

One in six ischaemic stroke patients has an embolic stroke of undetermined source (ESUS), defined as a stroke with unclear aetiology despite recommended diagnostic evaluation. The overall cardiovascular risk of ESUS is high and it is important to optimize strategies to prevent recurrent stroke and other cardiovascular events. The aim of clinicians when confronted with a patient not only with ESUS but also with any other medical condition of unclear aetiology is to identify the actual cause amongst a list of potential differential diagnoses, in order to optimize secondary prevention. However, specifically in ESUS, this may be challenging as multiple potential thromboembolic sources frequently coexist. Also, it can be delusively reassuring because despite the implementation of specific treatments for the individual pathology presumed to be the actual thromboembolic source, patients can still be vulnerable to stroke and other cardiovascular events caused by other pathologies already identified during the index diagnostic evaluation but whose thromboembolic potential was underestimated. Therefore, rather than trying to presume which particular mechanism is the actual embolic source in an ESUS patient, it is important to assess the overall thromboembolic risk of the patient through synthesis of the individual risks linked to all pathologies present, regardless if presumed causally associated or not. In this paper, a multi-disciplinary panel of clinicians/researchers from various backgrounds of expertise and specialties (cardiology, internal medicine, neurology, radiology and vascular surgery) proposes a comprehensive multi-dimensional assessment of the overall thromboembolic risk in ESUS patients through the composition of individual risks associated with all prevalent pathologies.

## Graphical Abstract

**Graphical Abstract:**
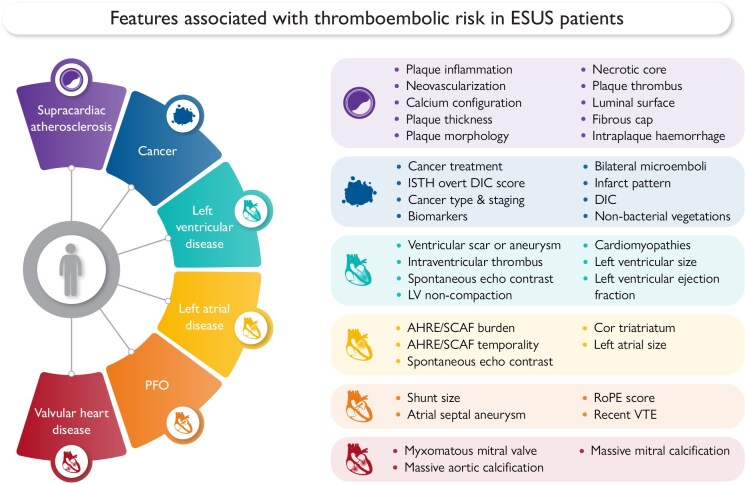
AHRE, atrial high-rate episode; DIC, disseminated intravascular coagulation; ISHT, International Society on Thrombosis and Haemostasis; LV, left ventricular; RoPE, Risk of Paradoxical Embolism; SCAF, subclinical atrial fibrillation; VTE, venous thromboembolism.

## Introduction

**Figure 1:**
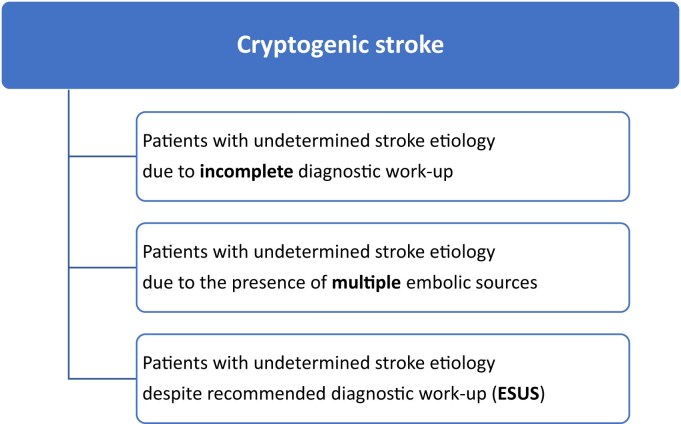
Distinction between the terms *cryptogenic stroke* and *embolic stroke of undetermined source*. ESUS, embolic stroke of undetermined source

One in six patients with ischaemic stroke has an embolic stroke of undetermined source (ESUS), defined as a stroke that despite recommended diagnostic workup, a convincing underlying cause like atrial fibrillation (AF), atherosclerotic plaque with high-grade stenosis, and others could not be identified.^1–3^ The term ESUS is not synonymous with the term ‘cryptogenic stroke’, as the latter also includes patients with multiple aetiologies, such as patients with both AF and atherosclerotic carotid stenosis ipsilateral to the infarct, and it also includes patients with incomplete diagnostic workup^4^ (*Figure 1*). The introduction of the ESUS concept in 2014 was based on explicit definition and diagnostic criteria, facilitated clinical research, and was widely adopted in clinical practice.^1,5^ Recently, an update of the ESUS criteria and diagnostic algorithm was published.^5^

Several pathologies can be the source of embolism in an ESUS patient, and they can be broadly categorized into supracardiac atherosclerosis, patent foramen ovale (PFO), and other right-to-left shunts, left atrial (LA) disease (including atrial arrhythmias and atrial cardiomyopathy), left ventricular (LV) disease, valvular heart disease, and cancer.^1–4^ The overall cardiovascular risk of ESUS patients is high, and their 5-year cumulative probability for stroke recurrence and other cardiovascular events is 29% and 38%, respectively, which highlights the importance of optimizing secondary preventive strategies to prevent recurrent strokes and other cardiovascular outcomes.^3^

The aim of any physician, when confronted with a patient not only with ESUS but also with any other medical condition of unclear aetiology, is to identify the *actual* cause amongst a list of potential differential diagnoses, in order to optimize strategies of treatment and prevention. However, specifically in patients with ESUS, this may be challenging and delusively reassuring.^6^ It can be *challenging* because in the majority of ESUS patients, multiple overlapping potential embolic sources exist, and it is frequently unclear which one was the actual embolic source.^7,8^ Also, it can be *delusively reassuring* because despite the establishment of specific secondary preventive measures for the individual pathology presumed to be the actual embolic source, a patient can still be vulnerable to future stroke and other cardiovascular events caused by other pathologies that were already present during the ESUS diagnostic workup but their thromboembolic potential was underestimated. In this context, rather than trying to presume which particular mechanism is the actual embolic source in an ESUS patient, it is of utmost importance to assess the *overall* thromboembolic risk of the patient through the synthesis of the individual risks linked to all prevalent pathologies, regardless if they are deemed to be causally associated with ESUS or not. A paradigm shift is justified, away from the assumption-based and inherently uncertain diagnostic label of *embolic stroke due to [presumed potential cause]* to the more comprehensive diagnostic term of *embolic stroke in a patient with [all potential causes]*.^6^ This can facilitate an integrated comprehensive approach to secondary prevention through the initiation of a bundle of therapeutic strategies according to the findings of the diagnostic workup, thereby optimizing the odds of achieving good patient outcomes.

In this position paper by the European Society of Cardiology (ESC) Council on Stroke in collaboration with the European Association of Cardiovascular Imaging and the European Heart Rhythm Association of the ESC, a multi-disciplinary panel of clinicians/researchers from various backgrounds of expertise and specialties (cardiology, internal medicine, neurology, radiology, and vascular surgery) proposes a comprehensive multi-dimensional assessment of the overall thromboembolic risk in patients with ESUS through the composition of individual risks associated with each of the six aforementioned broad pathologies (*Graphical Abstract*). It is emphasized that these are based largely on expert opinion and are summarized in *Table 1*. In addition, we discuss the potential implications for clinical practice and research and identify key knowledge gaps that should be addressed in future research.

**Table 1:** Features to assess the overall thromboembolic risk in embolic stroke of undetermined source patients

	Moderately increased thromboembolic risk	Slightly increased thromboembolic risk
Supracardiac atherosclerosis	Acute or recent intra-plaque haemorrhage Thin/ruptured fibrous cap Intraluminal thrombus Ulcerated plaque morphology	Lipid-rich necrotic core > 40% of the wall area. Maximum plaque thickness of the non-calcified component > 3 mm Irregular plaque morphology Positive rim sign Healed ulcerated plaque
Patent foramen ovale (*Figure 3*)	Recent DVT or pulmonary embolism AND presence of ASA or large shunt, regardless of patient age or RoPE score Presence of ASA or large shunt AND RoPE ≥ 7 and age < 60 years	Presence of ASA or large shunt AND RoPE < 7, regardless of patient age Absence of ASA or large shunt AND RoPE ≥ 7, regardless of patient age Recent DVT or pulmonary embolism, AND absence of ASA or large shunt, regardless of patient age or RoPE score
Left atrial disease	LA spontaneous echocardiographic contrast Cor triatriatum	
*Atrial arrhythmias*	AHRE or SCAF lasting >24 h and occurring within a month after stroke Any episode of atrial fibrillation lasting >30 s during stroke unit telemetry.	AHRE or SCAF lasting <24 h and occurring later than a month after stroke
*Atrial cardiomyopathy*		Significant LA enlargement (LA diameter > 4.6 cm)
Left ventricular disease	large or dyskinetic scar tissue LV aneurysm LV non-compaction with deep trabeculations or spongious LV Severe restrictive cardiomyopathy with decrease in cardiac output despite a preserved LV ejection fraction, e.g. cardiac amyloidosis.	Moderately decreased LV systolic function and/or very enlarged LV with spontaneous echo-contrast Cardiomyopathies, e.g. hypertrophic cardiomyopathy or amyloidosis Acute phase of an ischaemic cardiopathy
Valvular heart disease		Large redundant Barlow mitral valve disease Massive mitral valve calcifications Aortic valve calcifications
Cancer	Cardiac vegetation(s) with negative blood cultures ISTH overt DIC score ≥5 with D-dimer > 4000 ng/mL AND fibrinogen < 100 mg/dL AND platelet count < 100 10^3^/µL (OR PT prolongation > 3 s)	Cancer spread beyond primary site OR recent progression of disease OR centrally located primary or metastatic lung cancer D-dimer > 2500 ng/mL in the absence of an acute DVT or pulmonary embolism Acute infarction in multiple cerebral arterial territories Bilateral high-intensity transient signals on transcranial Doppler in the absence of a known central embolic source

AHRE, atrial high-rate episode; ASA, atrial septal aneurysm; DIC, disseminated intravascular coagulation; DVT, deep venous thrombosis; ISTH, International Society on Thrombosis and Haemostasis; LA, left atrial; LV, left ventricle; PFO, patent foramen ovale; RoPE, Risk of Paradoxical Embolism Score; SCAF, subclinical atrial fibrillation.

## Supracardiac atherosclerosis

The degree of carotid stenosis is considered the lead parameter for the assessment of a causal relation between the atherosclerotic process and an ischaemic event and for the prediction of the risk of stroke or transient ischaemic attack (TIA). This association was demonstrated >40 years ago in trials that showed the benefit of revascularization in patients with severe stenosis.^9^ At that time, the degree of arterial stenosis was the only characteristic of the plaque that could be assessed *in vivo* with angiography. Nowadays, imaging technology not only can visualize the entire supracardiac arterial tree but can also provide detailed information about the structure and composition of the plaque and its potential risk of rupture.^10^ Specific features of the plaque are associated with the risk of ischaemic events, regardless of the degree of stenosis.^11^ The prevalence of complex carotid artery plaque in patients with ESUS is more than five times higher ipsilateral to the infarct than contralateral.^12–14^ Also, the prevalence of complex carotid artery plaque in ESUS is two times higher compared with cardioembolic or small vessel stroke.^15^ These findings underline the important association between the risk of ischaemic events and the vulnerability of the plaque, not only in ESUS patients but generally in any patient with carotid plaque, which can be assessed by several parameters, as follows.

*Intra-plaque haemorrhage* (IPH) is defined by the accumulation of blood components within the atheromatous plaque and is considered the most important feature of plaque vulnerability. In a meta-analysis of seven cohort studies including 560 patients with symptomatic carotid stenosis and 136 patients with asymptomatic carotid stenosis, IPH was a stronger predictor of stroke than any known clinical risk factor. Amongst patients with a <50% stenosis, the annualized rates of ipsilateral stroke were 9.0% amongst patients with IPH and 0.7% amongst patients without.^16^ With magnetic resonance imaging (MRI), which is the best imaging technique for its detection, IPH can be classified according to its time of formation, either as fresh/acute (<1 week), recent (1–6 weeks), or remote/old (>6 weeks).^17^ The risk of cerebrovascular events is highest with acute/recent IPH but remains elevated for more than 18 months.^18^

The *fibrous cap* (FC), a layer of fibrous connective tissue that separates the core of the plaque from the arterial lumen, is another feature for the assessment of plaque vulnerability, preferably in MRI. A longitudinal prospective MRI-based study in 126 patients with symptomatic carotid stenosis who were followed for an average of 1 year showed that thin and/or ruptured FC was significantly associated with cerebrovascular events.^19^ Also, a thin and/or ruptured FC is highly associated with a recent stroke/TIA. For example, in an MRI-based study, patients with ruptured FC were 23 times more likely to have had a recent stroke/TIA compared with patients with thick FC.^20^ In another prospective longitudinal study, the disruption of the FC was a risk factor for cerebrovascular events.^21^

Another feature of vulnerability is the presence of carotid *intraluminal thrombus*, which presents with neurologic symptoms in up to 92% of cases.^22^ In a retrospective cross-sectional study of 726 carotid-brain MRI examinations, the strongest predictor of a carotid-source stroke was intraluminal thrombus followed by IPH.^23^ In another computed tomography angiography (CTA)-based study on 673 patients, the presence of intraluminal thrombi was highly predictive of the symptomatic side in carotid disease.^24^

The *luminal surface* of carotid plaques can be classified as smooth, irregular, or ulcerated.^25^ A smooth surface is identified as plain luminal morphology without any sign of ulceration or irregularity. An irregular surface is identified as the presence of small alterations of the luminal surface of the plaque, and its association with cerebrovascular events was described in the Northern Manhattan Study.^26^ An ulcerated surface is defined as an intimal defect that is >1 mm in width and exposes the necrotic core of the atheromatous plaque,^27^ and its association with cerebrovascular events is well established.^28–30^

The *lipid-rich necrotic core* (LRNC) is a heterogeneous tissue composed, amongst others, of cholesterol crystal, debris of apoptotic cells, and particles of calcium. The LRNC, preferably assessed in MRI, plays a key role in the progression and vulnerability of atherosclerotic plaques. A study of 120 asymptomatic patients with carotid plaque showed that a large LRNC size correlates with ipsilateral cerebrovascular events and that a LRNC > 40% of the wall area was prone to FC rupture in comparison with patients with a LRNC < 40%.^31^ In another study of 62 patients, a LRNC within the plaque strongly predicted cerebrovascular events.^32^ A systematic review and meta-analysis reported similar results showing that LRNC predicted stroke/TIA in asymptomatic subjects.^33^

Another important feature of plaque vulnerability is the *maximum plaque thickness* (MPT). This is distinct from stenosis as a thick plaque can occur without necessarily causing severe narrowing of the lumen. In a CTA-based study of 85 ESUS patients, >3 mm plaque thickness of the non-calcified component was present ipsilateral to stroke in 35% of patients and contralateral in 15%.^34^ In an MRI cross-sectional study of 1072 subjects, the MPT was more strongly associated with cerebral ischaemic symptoms than was the degree of stenosis.^35^

The role of calcium in atherosclerosis remains controversial, but it is generally agreed that the size, shape, and position of calcification may all affect plaque development.^36^ Amongst the different types of calcium configuration, the *positive rim sign*, defined as the presence of thin (<2 mm) adventitial calcifications with internal low attenuation plaque of ≥2 mm in maximum thickness in CTA, is associated with the presence of IPH.^37,38^ On the other hand, other calcium configurations such as intimal or superficial calcifications, or type 3, deep, or bulky calcifications, are rarely associated with the occurrence of cerebrovascular events.^39^

Intra-plaque *neovascularization* can be detected with contrast-enhanced ultrasound (CEUS), computed tomography (CT) and MRI, and requires the administration of contrast media for the detection and quantification of neovascularization.^40^ It can be seen in advanced atherosclerotic lesions, but, currently, routine clinical practice has not adopted non-invasive imaging assessment of plaque inflammation and neovascularization due to limitations related to the technical complexity of this analysis. A prospective CEUS study of 155 patients showed that intraplaque neovascularization is associated with recurrent cerebrovascular events regardless of the severity of carotid stenosis.^41^ An MRI study showed that adventitial enhancement, a marker of neovascularization, was associated with cerebrovascular events.^42^ The importance of wall enhancement as an additional marker of stroke risk stratification was also highlighted using CT.^43^ Currently, it is also possible to explore the level of *inflammation* within the plaque mainly with fluorodeoxyglucose positron emission tomography,^44^ but this parameter is also not currently adopted as a routine non-invasive imaging assessment.

Recently, the Carotid Plaque-RADS stroke risk classification system was introduced, which offers a morphological assessment of the thromboembolic risk associated with carotid plaques in addition to the prevailing quantitative parameter of stenosis.^45^

**Figure 2:**
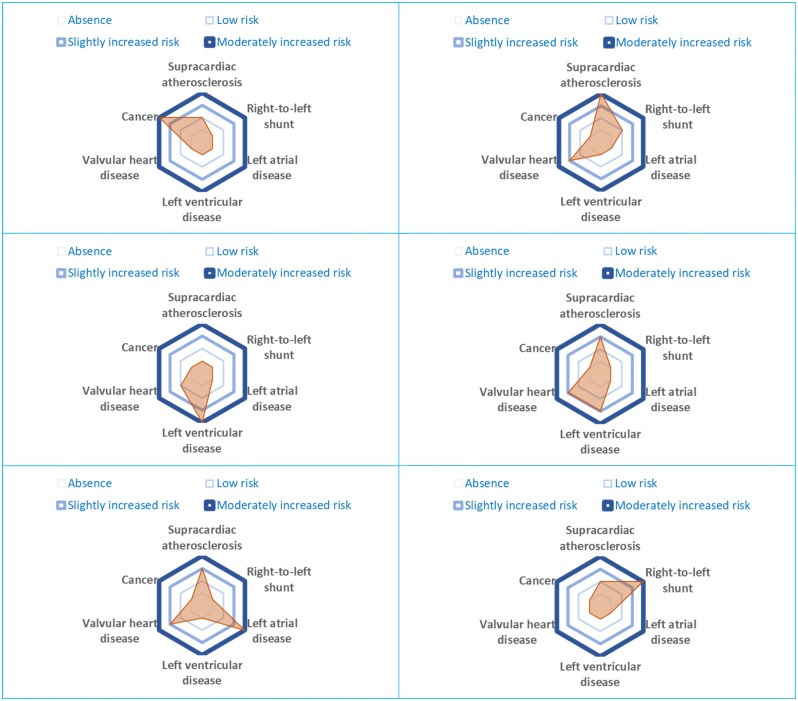
Examples of graphical illustration of the overall thromboembolic risk in embolic stroke of undetermined source patients. (Left upper panel) Patient with embolic stroke of undetermined source and metastatic lung cancer and vegetations at the aortic valve with sterile cultures and an ipsilateral-to-the-infarct atherosclerotic plaque in the common carotid artery causing 40% stenosis with smooth plaque surface and thick fibrous cap. (Right upper panel) A 68-year-old patient with embolic stroke of undetermined source and an ipsilateral-to-the-infarct atherosclerotic plaque in the internal carotid artery causing 40% stenosis with acute intra-plaque haemorrhage on magnetic resonance imaging; aortic valve calcification; and a patent foramen ovale with small shunt and no atrial septal aneurysm. (Left middle panel) Patient with embolic stroke of undetermined source and coronary artery disease with a large dyskinetic scar tissue in the left ventricular wall due to previous myocardial infarction and aortic valve stenosis with thin leaflets and no calcification. (Right middle panel) Patient with embolic stroke of undetermined source and an ipsilateral-to-the-infarct atherosclerotic plaque in the carotid bulb causing 40% stenosis with thick fibrous cap and irregular plaque morphology, but without intra-plaque haemorrhage or superimposed thrombus; heart failure with left ventricular ejection fraction 40%; and a large redundant Barlow mitral valve disease. (Left lower panel) patient with embolic stroke of undetermined source and spontaneous echocardiographic contrast in the left atrium; an ipsilateral-to-the-infarct non-ulcerated atherosclerotic plaque in the common carotid artery with thick plaque, lipid-rich necrotic core > 40% of the wall area without intra-plaque haemorrhage, or superimposed thrombus; and a calcified aortic valve. (Right lower panel) A 56-year-old patient with embolic stroke of undetermined source and a patent foramen ovale with atrial septal aneurysm and Risk of Paradoxical Embolism score 7 and an ipsilateral-to-the-infarct atherosclerotic plaque in the internal carotid artery causing 30% stenosis with significant calcification

**Figure 3:**
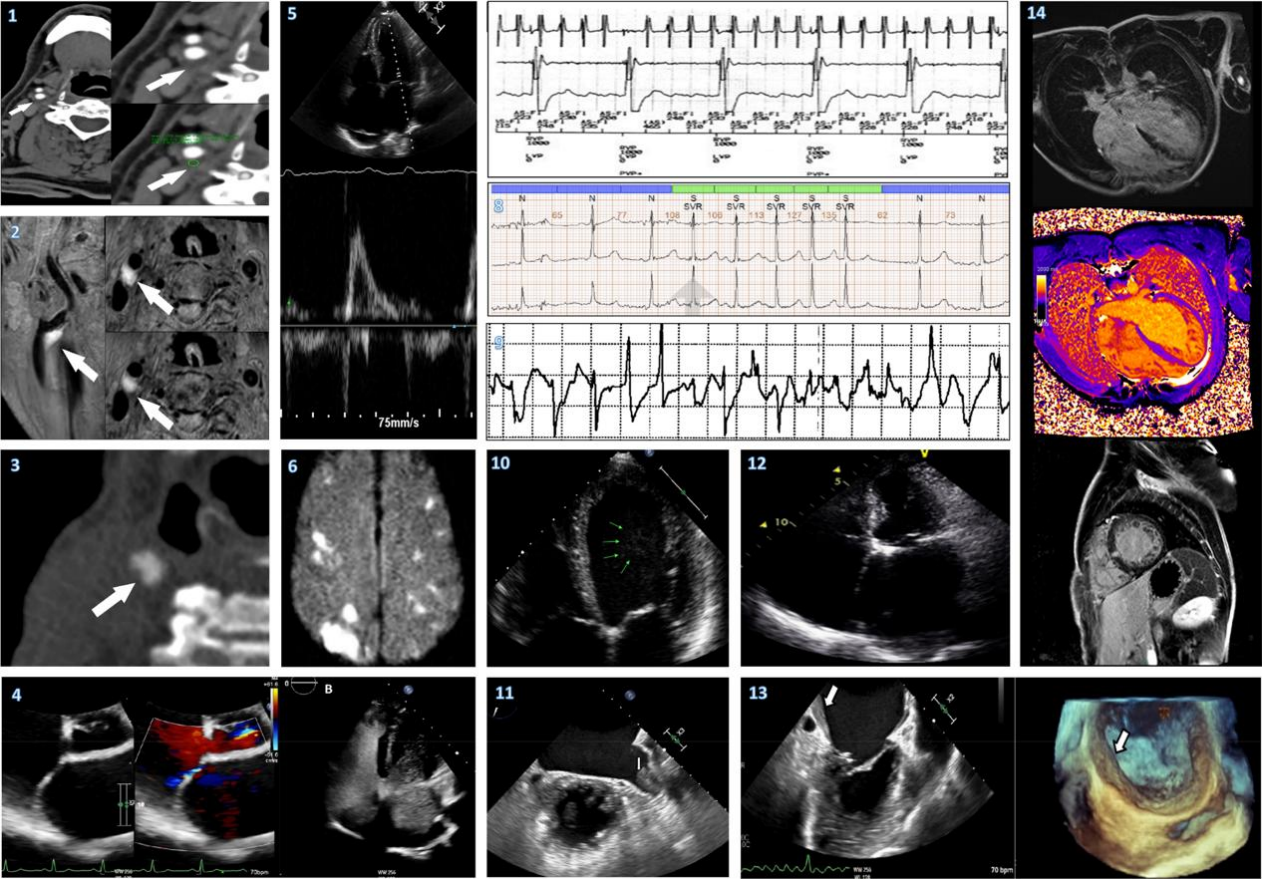
Features associated with thromboembolic risk in embolic stroke of undetermined source patients. (Panel 1) Computed tomography angiography axial scan of the right internal carotid artery in a 73-year-old symptomatic male patient with a 45% stenosis according to the NASCET criteria. A hypodense plaque is visible (arrow) with very low attenuation (<30 Hounsfield units). (Panel 2) MR shows intra-plaque haemorrhage at the bifurcation of the right carotid artery in a 75-year-old symptomatic male patient. (Panel 3) Computed tomography angiography axial scan of the right common carotid artery with a floating thrombus in a 69-year-old female patient. (Panel 4) Patent foramen ovale. (Left) Aneurysm of inter-atrial septum with small inter-atrial shunt in colour Doppler (transthoracic echocardiography, parasternal short-axis view). (Right) Massive right-to-left interatrial shunt during contrast echo with Valsalva manoeuvre. (Panel 5) A mitral inflow Doppler in a restrictive cardiomyopathy (amyloid) showing restrictive physiology, *E*/*A* > 3, Deceleration time < 120 ms and a short isovolumetric relaxation time. (Panel 6) Brain infarcts in all three cerebral arterial territories (three-territory sign) in a patient with advanced ceacal adenocarcinoma. (Panel 7) Pacemaker recording of an episode of atrial flutter with ventricular pacing. From top to bottom, tracings show atrial electrogram, ventricular electrogram, surface electrocardiogram (ECG), and pacemaker marker channels. (Panel 8) Surface electrocardiographic recording of a short run of non-sustained atrial tachycardia. (Panel 9) Pacemaker atrial electrogram showing an irregular atrial high-rate episode. (Panel 10) Spontaneous echo contrast in the left ventricle (arrows) of a patient with known dilated cardiomyopathy and left ventricular ejection fraction of 12%. (Panel 11) Thrombus (arrow) of the left atrial appendage in a patient with rheumatic mitral stenosis in transoesophageal echocardiography. (Panel 12) Echocardiography demonstrating a severely dilated left atrium in an ESUS patient without known atrial fibrillation. Reprinted from Kamel *et al*.^46^ with permission. (Panel 13) Extensive parietal thrombus of left atrium (arrow) in a patient with rheumatic mitral stenosis at 2D transoesophageal echocardiography (left) and 3D transoesophageal echocardiography (right). (Panel 14) Left ventricular non compaction at four-channel late gadolinium enhancement, T1 mapping, and short-axis view in cardiac magnetic resonance imaging

The proposed features to assess the thromboembolic risk in patients with ESUS and supracardiac atherosclerosis are presented in *Table 1*, and patient cases are illustrated in *Figures 2* and *3*.

We note that this section focuses and discusses the embolic source *per se* (i.e. the atherosclerotic plaque) and not the pathophysiologic entities that contribute to it like diabetes mellitus, arterial hypertension, smoking, dyslipidaemia, and others. The risk assessment and management of these pathologies are covered extensively in the related ESC Clinical Practice Guidelines. This is the case also for the next sessions of this Clinical Consensus Statement.

## Patent foramen ovale and other right-to-left shunts

Stroke due to paradoxical embolism may occur when a thrombus traverses a right-to-left shunt from the venous circulation into the arterial system and subsequently causes occlusion and infarction. The most common right-to-left shunt associated with stroke is PFO, but also other pathologies may exist such as other septal defects or intrapulmonary shunts. Alternatively, the thrombus may be formed *de novo* within the PFO tunnel. Epidemiologic studies have demonstrated a consistent relationship between ischaemic stroke and PFO in patients without another identifiable stroke aetiology. However, as PFO is a relatively common congenital anomaly found in about one-quarter of adults, its pathogenic role in stroke was contested for decades. Strokes occurring in patients with PFO were labelled as cryptogenic owing to this uncertainty, and the implementation of ESUS criteria in 2014 maintained the status of PFO as an undetermined source.^1^ Since 2017, several trials demonstrated that percutaneous closure of PFO reduced the risk of subsequent stroke amongst patients who otherwise met ESUS criteria.^47^ A meta-analysis using individual patient data from all trials reported a hazard ratio (HR) of 0.41 [95% confidence interval (CI) 0.28–0.60] for closure compared with medical therapy alone.^48^

These persuasive efficacy data underline that PFO can have significant thromboembolic risk in ESUS patients, the magnitude of which can be further evaluated by clinical and anatomic data, as well as clinical judgement after a thorough diagnostic workup.^49^ A classification has been proposed using the PFO-Associated Stroke CAusal Likelihood (PASCAL) algorithm.^50^ PASCAL combines the Risk of Paradoxical Embolism (RoPE) score, based on clinical characteristics, with key cardiac anatomic features of the PFO such as large right-to-left shunt and atrial septal aneurysm, both of which are associated with increased stroke risk^51^ and with signs of concurrent venous thromboembolism.^50^

The PASCAL algorithm was initially proposed based on inferences from epidemiology and pathophysiology, but the aforementioned individual patient data meta-analysis provided compelling retrospective validation.^50^ However, PASCAL was derived solely from clinical trial populations, nearly all patients were <60 years, and it has not been prospectively validated. The relative efficacy of closure compared with medical therapy alone was greater for patients in the PASCAL category ‘probably related’ (HR 0.10; 95% CI 0.03–0.35) compared with the category ‘possibly related’ (HR 0.38; 95% CI 0.22–0.65), and no benefit was observed for those in the category ‘relation unlikely’ (HR 1.14; 95% CI 0.53–2.46).^50^ The PASCAL classification is a rational and practical tool to aid in clinical decision-making. There are many other factors that have been reported to be associated with a causal role of PFO, including clinical features such as deep vein thrombosis, pulmonary or systemic embolism occurring close to the ischaemic stroke (within 48–72 h of stroke onset), stroke occurring at awakening in patients with sleep apnoea, circumstances that promote venous thrombotic events (e.g. prolonged travel, dehydration, or hypercoagulable state), stroke onset coincident with a Valsalva manoeuvre, a permanently increased right-to-left pressure gradient (due to chronic arterial pulmonary hypertension or right heart diseases), a history of non-cerebral embolism, a history of migraine with aura, and decompression illness, and anatomic features including the presence of a Eustachian valve or Chiari network, but these were not integrated into the PASCAL tool and require further research. Also, there is no quality evidence to support that ESUS patients should have venous ultrasound to detect potential embolic sources.

In patients < 60 years with ‘possible’ or ‘probable’ PFO-related stroke according to PASCAL who have no other probable embolic source, the PFO could be considered as the actual cause of stroke and hence, not considered as an ESUS. Accordingly, this is also proposed in the updated ESUS criteria.^5^ Moreover, the role of other simultaneously additional or alternative pathways for right-to-left shunt, including other cardiac septal defects or pulmonary arteriovenous malformations, deserves further investigation.

**Figure 4:**
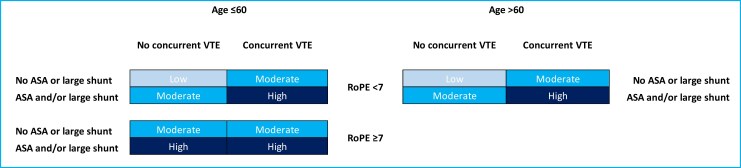
Assessment of thromboembolic risk related to patent foramen ovale in embolic stroke of undetermined source patients based on the morphological features of the patent foramen ovale, the Risk of Paradoxical Embolism score, patient age, and history of recent venous thromboembolism. The right lower part of the figure is blank because the maximum Risk of Paradoxical Embolism score that a patient > 60 years can have is 6. ASA, atrial septal aneurysm; VTE, venous thromboembolism

Therefore, the proposed criteria for assessing the thromboembolic risk in patients with ESUS and PFO expand beyond the PASCAL classification, which largely applies to younger patients and does not include all related clinical features. Instead, a broader set of criteria also including age and venous thrombotic events is proposed in *Table 1* and *Figure 4*, and patient cases are illustrated in *Figures 2* and *3*.

## Left atrial disease

The presence of thrombus in the LA or the LA appendage (LAA) carries a very high embolic risk.^52^ Thrombi have a prevalence of approximately 3% in anticoagulated persons with AF,^53^ which is higher in non-paroxysmal than in paroxysmal AF (approximately 4.8% and 1%, respectively).^53^ The risk is even higher, approximately 9%, in non-anticoagulated persons with AF.^54^ Conversely, in persons without an AF diagnosis, its prevalence is low but can be associated with mitral valve disease,^55^ atrial cardiomyopathy,^55,56^ or covert AF. In a patient with stroke, the presence of LA thrombus is a convincing aetiology of cardioembolic stroke, and such patients should not be classified as ESUS. *Spontaneous echocardiographic contrast* in the LA is the cardiac factor which is most strongly related to LAA thrombus.^57^

*Cor triatriatum sinistrum* is a congenital cardiac anomaly in which the LA is divided into two chambers by a membrane. It is a rare finding as it represents only 0.4% of all congenital heart defects.^58^ Given its rarity, finding a cor triatriatum sinistrum in a patient with ESUS should be regarded as a probable stroke aetiology.

### Atrial arrhythmias

The strong association of AF with thromboembolic events in observational studies, coupled with the profound effect of oral anticoagulation (OAC) on stroke prevention in persons with AF, provided undisputed evidence about the role of AF in stroke.^59^ However, it should be emphasized that the beneficial role of OAC is proven only in AF detected with the standard 12-lead ECG (frequently termed as clinical AF) and not AF detected after stroke (frequently termed as AFDAS) such as AF detected during telemetry at the stroke unit or during non-invasive monitoring like Holter ECG or 14-day patch ECG soon after stroke. New-onset AF is identified in approximately 13% of stroke patients during the in-hospital phase,^60^ and such patients should not be classified as ESUS.^1^ More episodes of asymptomatic AF may be detected in the outpatient setting with the use of cardiac rhythm monitoring devices.^60^ With the use of prolonged, continuous cardiac monitoring in patients following stroke, short-lasting episodes (minutes to hours) of asymptomatic AF with a very low burden of AF (defined as <1% of AF over monitoring time) are often detected, and this entity is termed as *subclinical atrial fibrillation* (SCAF) or *atrial high-rate episodes* (AHRE) if ECG/electrograms are not available.^61^ The risk of stroke is higher in persons with SCAF/AHRE than in patients without, but lower than in patients with clinical AF.^61,62^ In the non-anticoagulated groups of the ARTESIA and NOAH-AFNET 6 trials, the rate of ischaemic stroke was 1.1–1.2/100 person-years.^62,63^ It seems plausible that a causal association between SCAF/AHRE and ESUS is stronger in patients with episodes that last longer (e.g. >24 h^64,65^) or occur proximal to the stroke,^64,66^ rather than in patients with shorter episodes occurring distally. The role of OAC in individuals with SCAF/AHRE was recently tested in the ARTESIA and NOAH-AFNET 6 trials.^62,63^ In the study-level meta-analysis of these two trials, there was significant reduction in the rate of ischaemic stroke with OAC [relative risk (RR) 0.68; 95% CI 0.50–0.92, with no sign of heterogeneity) and significant increase in the rate of major bleeding (RR 1.62; 95% CI 1.05–2.5).^67^ The duration of AHRE did not interact with the efficacy or safety of anticoagulation, either using the 24-h threshold or as a continuous covariate in the NOAH-AFNET 6 trial.^68^ Only 9.4% of included patients had previous stroke/TIA or systemic embolism. Further analyses of the trials are warranted to identify which parameters, clinical and non-clinical (like the burden of arrhythmia, imaging findings, and biomarkers), can provide a better estimate of the risk of stroke.

### Atrial cardiomyopathy

The size of the LA has been consistently associated with a higher risk of AF^69^ and stroke.^70,71^  *Enlargement of the LA* may be associated with age-related progressive remodelling, stretch from pressure and volume overload, and oxidative stress and inflammation and could form an arrhythmogenic and thrombogenic milieu.^72,73^ The possible role of LA dilatation in ESUS is supported by a *post hoc* analysis of the NAVIGATE-ESUS trial that showed that oral rivaroxaban was associated with a reduced risk of recurrent stroke amongst patients with ESUS and a LA diameter of >4.6 cm.^74^ The ARCADIA trial assessed the role of OAC with apixaban in patients with ESUS and signs of atrial cardiopathy.^75^ The trial was stopped after its planned interim analysis for futility as there was no difference in stroke recurrence rate between apixaban- and aspirin-assigned patients.

The proposed features to assess the thromboembolic risk in patients with ESUS and LA disease are presented in *Table 1*, and patient cases are illustrated in *Figures 2* and *3*.

## Left ventricular disease

Left ventricular systolic dysfunction is commonly related to stroke, and it is important to diagnose LV disease (including heart failure with preserved ejection fraction) and initiate state-of-the-art heart failure therapy.^76^ Ischaemic heart disease and dilated cardiomyopathies with reduced LV ejection fraction (LVEF) are the most common causes of LV dysfunction which may cause stroke.^77^ Per the ESUS definition, patients with LVEF < 30% are not classified as ESUS given the well-established association between severely reduced LVEF and stroke. Several studies assessed the role of OAC in patients with reduced LVEF. The WARCEF trial reported that OAC in persons in sinus rhythm and severely reduced LVEF reduced the rate of ischaemic stroke compared with warfarin but increased the risk of major bleeding.^78^ Similar conclusions were drawn by a recent meta-analysis of all related trials in which OAC in persons with heart failure and sinus rhythm was associated with a 43% RR reduction of stroke or systemic embolism, which was offset by a 92% RR increase of major bleeding.^79^

Other forms of LV disease may be also the underlying source of ESUS. *Cardiac amyloidosis* can lead to severe restrictive cardiomyopathy with reduced cardiac output despite preserved LVEF and subsequent thrombus in the LV and also in the LA.^80^ Thrombus formation may develop in case of *large or dyskinetic scars* or *LV aneurysms* in the chronic phase of myocardial infarction, in *hypertrophic*, *LV non-compaction and other cardiomyopathies*, as well as in patients with *reduced LV function in valvular heart disease.*^81^ In patients with stroke and LV thrombus, the causal association should be regarded as definite cardioembolism and such patients should not be classified as ESUS. The management of LV thrombi was recently reviewed.^82^ Oral anticoagulation is recommended in patients with LV non-compaction cardiomyopathy and prior embolic event.^83^ Left ventricular *spontaneous echo-contrast* can be seen in patients with low cardiac output and is highly associated with the occurrence of thrombi and potential systemic emboli. The acute phase of ischaemic heart diseases is also associated with an increased risk of stroke.^84^ Although the incidence of LV thrombus after acute anterior myocardial infarction with new-onset wall motion abnormalities is low, an increased RR persists in the present era despite current revascularization strategies and dual antiplatelet therapy.^85^

The proposed features to assess the thromboembolic risk in patients with ESUS and LV disease are presented in *Table 1*, and patient cases are illustrated in *Figures 2* and *3*.

## Valvular heart disease

Prosthetic cardiac valves, mitral stenosis, and vegetations due to infective endocarditis are considered as major risk sources of cardioembolism, and in their presence, a patient with stroke should not be classified as ESUS.^1^ Beyond these entities, the association between valvular pathologies and stroke is weak. The native valvular heart diseases are rare sources of cardiac emboli if we exclude the specific context of endocarditis^86,87^ and cardiac tumours.^77,88^ In persons with AF, significant aortic valve disease and mitral regurgitation are associated with an increased risk of systemic embolism as compared with patients without valvular disease.^89^

Highly redundant myxomatous mitral valves (Barlow disease) and massive valvular calcifications are two valvular pathologies that are associated with increased embolic risk.^90^ These calcifications are becoming more prevalent.^91^ They are found on the aortic valve^92^ and also on the mitral valve, especially the posterior mitral valve leaflet and the annulus. The valvular calcifications can be associated with more diffuse atherosclerotic disease, such as complex aortic atheroma.^93^

Lambl’s excrescences, or else valvular strands, are thin filiform mobile processes that can be found in the mitral and aortic valves. Although it was hypothesized that they are associated with increased stroke risk, observational studies reported inconsistent results.^94,95^ In a recent analysis, Lambl’s excrescences were not associated with ESUS.^95^

The proposed features to assess the thromboembolic risk in patients with ESUS and valvular heart disease are presented in *Table 1*, and patient cases are illustrated in *Figures 2* and *3*.

## Cancer

Cancer is a complex disease that can cause ischaemic stroke through several mechanisms, including hypercoagulable processes such as non-bacterial thrombotic endocarditis (NBTE) and intravascular coagulation, direct vascular injury such as tumour emboli and external compression, and treatment complications such as radiation vasculopathy and chemotherapy-induced cardiomyopathy.^96,97^ In the year before cancer is diagnosed, there is a 59% increased risk of ischaemic stroke.^98^ Looking in reverse, 2%–10% of patients with ischaemic stroke are diagnosed with cancer in the subsequent year.^99–102^ Once cancer is diagnosed, stroke risk remains elevated and is doubled in the 6 months thereafter.^103^ The increased stroke risk in patients with cancer follows a U-shaped curve, with risks peaking near the time of cancer diagnosis and then many years later, and the later peak driven by cumulative deleterious effects of cancer treatments.^96^ In the USA, 14% of patients hospitalized with ischaemic stroke in 2019 had active or prior cancer.^104^ About 50% of cancer-related strokes are classified as ESUS after standard evaluation.^105^ Many cancer patients with strokes classified as ESUS have a distinctive phenotype with advanced or progressive cancer, very elevated clotting markers, and evidence for a central embolic process on imaging.^106^ These observations highlight the important link between cancer and stroke and raise the question of whether cancer-related stroke should be considered its own stroke subtype.

According to autopsy data from a cancer centre in the 1980s, NBTE is a leading cause of stroke in patients with cancer.^107^ Non-bacterial thrombotic endocarditis is characterized by sterile platelet-fibrin vegetations on cardiac valves. Vegetations are generally small and friable, and therefore, definitive diagnosis antemortem is rare, even with transoesophageal echocardiography.^108,109^ Immunohistochemical analyses of thrombectomy specimens have demonstrated similar clot profiles between cancer patients with non-infectious cardiac vegetations and those with ESUS, suggesting that NBTE is an underappreciated cause of cancer-related stroke.^110^

Advanced solid or haematological cancer can cause *disseminated intravascular coagulation* (DIC) leading to stroke. The International Society on Thrombosis and Haemostasis (ISTH) has established criteria, based on levels of D-dimer, fibrinogen, platelet count, and prothrombin time, to diagnose overt DIC in patients with malignancy.^111^ While D-dimer elevations are common in patients with cancer and stroke, overt DIC fulfilling ISTH criteria is rare.^97^

Embolic stroke of undetermined source in cancer patients often has a *distinctive clinical phenotype* with fewer prevalent vascular risk factors; cancer types associated with increased risks of thromboembolism (e.g. lung, pancreas, gastric, colorectal, ovarian, bladder, and non-Hodgkin’s lymphoma); cancers that are newly diagnosed, metastatic, or progressing through treatment; and multi-territory embolic-appearing infarct patterns.^105^ Cancer patients with ESUS also often have activation of multiple pathways promoting thrombosis, including platelets, coagulation factors, endothelium, neutrophil extracellular traps, and extracellular vesicles.^105,106,112^ The most consistent markers routinely available in practice are the multi-territorial infarct pattern and D-dimer.

Small, diffuse, predominantly cortical, acute infarcts involving multiple cerebral arterial territories occur in 30%–70% of patients with active cancer and ESUS.^106,113,114^ This infarct pattern, while not specific, can help identify previously occult cancer in patients with ESUS^100^ and is associated with an increased risk of early neurological deterioration.^115^ When involving all three cerebral arterial territories, it has been termed the *three-territory sign*.^116^

*Transcranial Doppler (TCD) high-intensity transient signals (HITS)*, surrogates for circulating microemboli, are present in 38%–58% of patients with cancer and ESUS, a rate much higher than in stroke patients without cancer, correlate with plasma D-dimer levels, and are associated with adenocarcinoma histology and an increased risk of recurrent stroke or death.^117,118^ If bilateral, HITS suggest a central embolic process.

Extreme elevations of thrombotic end-products such as *plasma D-dimer* are common in patients with cancer and ESUS.^100^ In a prospective study of 50 patients with solid cancer and acute ischaemic stroke, the median D-dimer value was 2.552 ng/mL, compared with median values of 405 and 670 ng/mL in matched patients with stroke only and cancer only, respectively.^106^ In this study, D-dimer levels were associated with an increased risk of recurrent stroke and death.^117^ The optimal threshold to identify an occult cancer or posit causation to a known cancer is uncertain, with reported cut-offs ranging from 820 to 10 000 ng/mL.^102,119,120^ We chose a cut-off of 2500 ng/mL for our proposed criteria, as it is close to most published cut-offs and is five times the upper limit of normal. It is worth noting that D-dimer is not specific and can be increased by thromboembolic events besides stroke, AF, prosthetic heart valves, trauma, surgery, and poor sample collection. Many other haematological, inflammatory, and tumour markers are also increased in cancer-related stroke, including P-selectin, thrombin–antithrombin, thrombomodulin, soluble intercellular adhesion molecule, vascular cellular adhesion molecule, CA19-9, CA125, and C-reactive peptide.^106,121,122^ However, the relative difference for these markers between cancer and non-cancer patients is not as large as for D-dimer, and many are costly and not routinely available in practice.^106^ It is emphasized that plasma D-dimer and TCD HITS are not specific to cancer as an ESUS aetiology but rather serve as quantitative surrogate markers for central embolic processes, which may contribute to ESUS, including atrial cardiomyopathy and atrial arrhythmias.

Tumour embolism is likely an under-recognized cause of stroke in patients with cancer. *Centrally located primary or metastatic lung cancers* can invade the pulmonary veins or cardiac chambers and embolize to the brain causing stroke.^123^ Patients with tumour embolism who survive long enough may develop metastases at the site of their stroke.

Nearly all cancer treatments have been associated with increased stroke risk, particularly platinum-based chemotherapy, tyrosine kinase inhibitors, vascular endothelial growth factor inhibitors, and hormonal therapy.^96^ However, these associations are often confounded by indication and disappear after adjustment for cancer status,^124^ and in most cases, cancer treatments are probably innocent bystanders or triggers for stroke and not the actual cause. The exceptions to this are surgery causing perioperative stroke through direct injury to the heart or cervicocephalic vessels and radiation causing delayed vasculopathy or heart disease, although in these scenarios, patients’ stroke mechanism is known.^125^ For this position paper, we have restricted thromboembolism risk to the cancer itself and not its treatments, which we believe are separate entities with differing characteristics and outcomes.

The criteria that we proposed for the assessment of thromboembolic risk amongst patients with cancer and ESUS should not affect management decisions, as the prothrombotic effects seen in cancer and stroke are multi-fold and involve platelets as much as coagulation factors, and bleeding risks in these patients are exceedingly high.^117^

The proposed features to assess the thromboembolic risk in patients with ESUS and cancer are presented in *Table 1*, and patient cases are illustrated in *Figures 2* and *3*.

## Diagnostic evaluation

It is evident that the complexity of ESUS precludes a one-size-fits-all approach in the diagnostic evaluation of patients. Diagnostic algorithms for ESUS have been proposed,^126^ but the large number of associated pathologies, the even larger number of risk factors that contribute to these pathologies, and the availability of several diagnostic methods to assess the heart and the arteries require an individualized approach. This should be guided by specific patient characteristics, scalable quantifiable proxies of these pathologies, as well as availability and expertise in imaging techniques. These could include age, comorbidities, and smoking/diet habits, lipid profile, and other organ-specific blood biomarkers as discussed throughout the previous sections, estimation of the cardiovascular risk of the patient using validated risk scores, estimation of the likelihood of covert AF using validated prognostic scores like the AF-ESUS score,^127,128^ vascular ultrasound, CT/CTA, and MRI/magnetic resonance angiography to visualize the arterial circulation and the heart, and others. This highly personalized integrated strategy warrants broad expertise in several fields and calls for clinicians who care for stroke patients to broaden their skills across these domains. We need stroke specialists to take ownership of all these facets, rather than just diffusing responsibility across a whole bunch of consulting specialists.^129^

## Implications for clinical practice and research

Patients with ESUS have a considerable risk for stroke recurrence and other cardiovascular events, which highlights the importance to optimize preventive strategies to mitigate this risk. For several decades, there were no clear criteria to define the term ‘cryptogenic stroke’, which hindered clinical research in this field. In 2014, the introduction of the ESUS concept with explicit definition and criteria led to an improved standardization of this population and hence potentiated clinical research with several randomized trials assessing the role of preventive strategies.^1^ The first intervention that was tested in the general ESUS population was OAC with direct oral anticoagulants. However, the results of three randomized trials were disappointing.^130–132^ Consequently, it was suggested that future research on ESUS should be tailored to patients with specific characteristics that point towards specific embolic sources.^46^ The present position paper has the potential to further enhance research in this population, as it can support a more accurate and homogeneous phenotypic clustering of patients according to their characteristics and consequently facilitate randomized trials of targeted preventive strategies.

## Key knowledge gaps

Further evidence is needed to optimize secondary prevention in ESUS. Future secondary prevention trials in ESUS could provide this, as it has been the case for trials of percutaneous PFO closure, which, through their positive results, provided practice-changing evidence for patients < 60 years. *Table 2* presents a list of open questions. For example, in patients with ESUS and supracardiac atherosclerosis, trials of intensive antithrombotic treatment (e.g. addition of a factor XIa inhibitor or low-dose rivaroxaban to standard antiplatelet treatment); intensive lipid-lowering treatment to more aggressive targets than currently recommended; anti-inflammatory drugs; lowering of lipoprotein(a); and intervention with endarterectomy or stenting are warranted. Also, in patients with ESUS and PFO who are >60 years of age, trials of percutaneous PFO closure are needed. In addition, for specific populations of patients with LA, LV disease, or with SCAF/AHRE, further trials of OAC are necessary. The minimal AF burden that merits OAC is unknown. Also, in patients with ESUS and cancer, trials of OAC alone or a combination of OAC and antiplatelet therapy are warranted.

**Table 2:** Key knowledge gaps

In patients with ESUS and supracardiac atherosclerosis, is intensive lipid-lowering treatment superior compared with standard of care for the prevention of stroke recurrence?
In patients with ESUS and supracardiac atherosclerosis, is intensive antithrombotic treatment superior compared with currently recommended antiplatelet treatment for the prevention of stroke recurrence?
In patients with ESUS and supracardiac atherosclerosis and increased levels of lipoprotein(a), is lowering of lipoprotein(a) beneficial for the prevention of stroke recurrence?
In patients with ESUS and supracardiac atherosclerosis and increased markers of inflammation, is anti-inflammatory treatment beneficial for the prevention of stroke recurrence?
In patients with ESUS and supracardiac atherosclerosis, is intervention with endarterectomy or stenting superior compared with medical-only treatment for the prevention of stroke recurrence?
In patients with ESUS and PFO who are >60 years of age, is percutaneous PFO closure superior compared with medical-only treatment for the prevention of stroke recurrence?
In patients with ESUS and LA spontaneous echo contrast, is OAC superior compared with antiplatelet treatment for the prevention of stroke recurrence?
In which subgroups of patients with AHRE/SCAF does the benefit of OAC clearly outweigh the harm of associated bleeding?
How can we improve the prediction of stroke risk in patients with AHRE/SCAF?
What is the optimal definition of AF burden?
What is the minimal AF burden which merits OAC in ESUS patients?
In patients with ESUS and significant LA enlargement, is OAC superior compared with antiplatelet treatment for the prevention of stroke recurrence?
In patients with ESUS and LV disease, is OAC superior compared with antiplatelet treatment for the prevention of stroke recurrence?
In patients with ESUS and valvular heart disease, is valvular intervention superior compared with medical-only treatment for the prevention of stroke recurrence?
In patients with ESUS and cancer, is OAC superior compared with antiplatelet treatment for the prevention of stroke recurrence?

AF, atrial fibrillation; AHRE, atrial high-rate episode; ESUS, embolic stroke of undetermined source; LA, left atrial; LV, left ventricle; OAC, oral anticoagulation; PFO, patent foramen ovale; SCAF, subclinical atrial fibrillation.

## Synthesis and concluding remarks

When the negative results of the trials of OAC compared with aspirin in the general ESUS population were published,^130–132^ criticism was addressed towards the ESUS concept. It was suggested that ESUS is not a useful concept and has failed. This is far from the truth: on the contrary, what has actually failed by the results of these trials is not the ESUS concept itself, but the hypothesis that OAC is beneficial in all of these patients and, consequently, the hypothesis that the majority of ESUS is due to covert AF. As clearly stated in the landmark paper that introduced ESUS,^1^ in the publication that introduced the updated ESUS criteria and diagnostic algorithm,^5^ in the present position paper and elsewhere,^4,46^ the underlying aetiologies in ESUS are numerous and frequently overlapping. The failure of the hypothesis that covert AF is a leading aetiology of ESUS as emphatically implied by the results of the NAVIGATE-ESUS^130^ and RE-SPECT ESUS^131^ trials, urges the research community to redirect focus on assessing the role of other preventive strategies in ESUS, as discussed above, which could potentially improve the prognosis in this patient population.

The high prevalence of ESUS, the considerable risk for stroke recurrence, the modern diagnostic imaging techniques, and the availability of several medical and interventional strategies that could potentially reduce stroke risk in ESUS-associated pathologies like supracardiac atherosclerosis, PFO, LA, or LV disease, valvular heart disease, and cancer identify ESUS as an important priority in stroke research in the coming years, which may hopefully improve outcomes in this large patient population.

## Data Availability

No data were generated or analysed for this manuscript.

## References

[1] Hart RG, Diener HC, Coutts SB, Easton JD, Granger CB, O'Donnell MJ, et al Embolic strokes of undetermined source: the case for a new clinical construct. Lancet Neurol 2014;13:429–38. 10.1016/S1474-4422(13)70310-7 24646875

[2] Hart RG, Catanese L, Perera KS, Ntaios G, Connolly SJ. Embolic stroke of undetermined source: a systematic review and clinical update. Stroke 2017;48:867–72. 10.1161/strokeaha.116.016414 28265016

[3] Ntaios G, Papavasileiou V, Milionis H, Makaritsis K, Manios E, Spengos K, et al Embolic strokes of undetermined source in the Athens stroke registry: a descriptive analysis. Stroke 2015;46:176–81. 10.1161/strokeaha.114.007240 25378429

[4] Ntaios G . Embolic stroke of undetermined source: JACC review topic of the week. J Am Coll Cardiol 2020;75:333–40. 10.1016/j.jacc.2019.11.024 31976872

[5] Diener HC, Easton JD, Hart RG, Kasner S, Kamel H, Ntaios G. Review and update of the concept of embolic stroke of undetermined source. Nat Rev Neurol 2022;18:455–65. 10.1038/s41582-022-00663-4 35538232

[6] Ntaios G, Hart RG. Embolic stroke. Circulation 2017;136:2403–5. 10.1161/circulationaha.117.030509 29255121

[7] Ntaios G, Pearce LA, Veltkamp R, Sharma M, Kasner SE, Korompoki E, et al Potential embolic sources and outcomes in embolic stroke of undetermined source in the NAVIGATE-ESUS trial. Stroke 2020;51:1797–804. 10.1161/STROKEAHA.119.028669 32295509

[8] Ntaios G, Perlepe K, Lambrou D, Sirimarco G, Strambo D, Eskandari A, et al Prevalence and overlap of potential embolic sources in patients with embolic stroke of undetermined source. J Am Heart Assoc 2019;8:e012858. 10.1161/jaha.119.012858 31364451 PMC6761628

[9] Rothwell PM, Eliasziw M, Gutnikov SA, Warlow CP, Barnett HJM. Endarterectomy for symptomatic carotid stenosis in relation to clinical subgroups and timing of surgery. Lancet 2004;363:915–24. doi: 10.1016/S0140-6736(04)15785-1 15043958

[10] Saba L, Saam T, Jager HR, Yuan C, Hatsukami TS, Saloner D, et al Imaging biomarkers of vulnerable carotid plaques for stroke risk prediction and their potential clinical implications. Lancet Neurol 2019;18:559–72. 10.1016/s1474-4422(19)30035-3 30954372

[11] Ntaios G, Wintermark M, Michel P. Supracardiac atherosclerosis in embolic stroke of undetermined source: the underestimated source. Eur Heart J 2021;42:1789–96. 10.1093/eurheartj/ehaa218 32300781

[12] Kamtchum-Tatuene J, Wilman A, Saqqur M, Shuaib A, Jickling GC. Carotid plaque with high-risk features in embolic stroke of undetermined source: systematic review and meta-analysis. Stroke 2020;51:311–4. 10.1161/strokeaha.119.027272 31752616 PMC6993880

[13] Ntaios G, Swaminathan B, Berkowitz SD, Gagliardi RJ, Lang W, Siegler JE, et al Efficacy and safety of rivaroxaban versus aspirin in embolic stroke of undetermined source and carotid atherosclerosis. Stroke 2019;50:2477–85. 10.1161/strokeaha.119.025168 31401971

[14] Ntaios G, Perlepe K, Sirimarco G, Strambo D, Eskandari A, Karagkiozi E, et al Carotid plaques and detection of atrial fibrillation in embolic stroke of undetermined source. Neurology 2019;92:e2644–52. 10.1212/wnl.0000000000007611 31068479

[15] Kopczak A, Schindler A, Bayer-Karpinska A, Koch ML, Sepp D, Zeller J, et al Complicated carotid artery plaques as a cause of cryptogenic stroke. J Am Coll Cardiol 2020;76:2212–22. 10.1016/j.jacc.2020.09.532 33153580

[16] Schindler A, Schinner R, Altaf N, Hosseini AA, Simpson RJ, Esposito-Bauer L, et al Prediction of stroke risk by detection of hemorrhage in carotid plaques: meta-analysis of individual patient data. JACC Cardiovasc Imaging 2020;13:395–406. 10.1016/j.jcmg.2019.03.028 31202755

[17] Lusby RJ, Ferrell LD, Ehrenfeld WK, Stoney RJ, Wylie EJ. Carotid plaque hemorrhage. Its role in production of cerebral ischemia. Arch Surg 1982;117:1479–88. 10.1001/archsurg.1982.01380350069010 6182861

[18] Takaya N, Yuan C, Chu B, Saam T, Polissar NL, Jarvik GP, et al Presence of intraplaque hemorrhage stimulates progression of carotid atherosclerotic plaques: a high-resolution magnetic resonance imaging study. Circulation 2005;111:2768–75. 10.1161/CIRCULATIONAHA.104.504167 15911695

[19] Kwee RM, van Oostenbrugge RJ, Mess WH, Prins MH, van der Geest RJ, ter Berg JW, et al MRI of carotid atherosclerosis to identify TIA and stroke patients who are at risk of a recurrence. J Magn Reson Imaging 2013;37:1189–94. 10.1002/jmri.23918 23166040

[20] Yuan C, Zhang SX, Polissar NL, Echelard D, Ortiz G, Davis JW, et al Identification of fibrous cap rupture with magnetic resonance imaging is highly associated with recent transient ischemic attack or stroke. Circulation 2002;105:181–5. 10.1161/hc0202.102121 11790698

[21] Sadat U, Teng Z, Young VE, Walsh SR, Li ZY, Graves MJ, et al Association between biomechanical structural stresses of atherosclerotic carotid plaques and subsequent ischaemic cerebrovascular events–a longitudinal in vivo magnetic resonance imaging-based finite element study. Eur J Vasc Endovasc Surg 2010;40:485–91. 10.1016/j.ejvs.2010.07.015 20724181

[22] Bhatti AF, Leon LR Jr, Labropoulos N, Rubinas TL, Rodriguez H, Kalman PG, et al Free-floating thrombus of the carotid artery: literature review and case reports. J Vasc Surg 2007;45:199–205. 10.1016/j.jvs.2006.09.057 17210411

[23] McNally JS, McLaughlin MS, Hinckley PJ, Treiman SM, Stoddard GJ, Parker DL, et al Intraluminal thrombus, intraplaque hemorrhage, plaque thickness, and current smoking optimally predict carotid stroke. Stroke 2015;46:84–90. 10.1161/STROKEAHA.114.006286 25406146

[24] Eesa M, Hill MD, Al-Khathaami A, Al-Zawahmah M, Sharma P, Menon BK, et al Role of CT angiographic plaque morphologic characteristics in addition to stenosis in predicting the symptomatic side in carotid artery disease. AJNR Am J Neuroradiol 2010;31:1254–60. 10.3174/ajnr.A2078 20360336 PMC7965448

[25] Saba L, Anzidei M, Marincola BC, Piga M, Raz E, Bassareo PP, et al Imaging of the carotid artery vulnerable plaque. Cardiovasc Intervent Radiol 2014;37:572–85. 10.1007/s00270-013-0711-2 23912494

[26] Prabhakaran S, Rundek T, Ramas R, Elkind MS, Paik MC, Boden-Albala B, et al Carotid plaque surface irregularity predicts ischemic stroke: the northern Manhattan study. Stroke 2006;37:2696–701. 10.1161/01.STR.0000244780.82190.a4 17008627 PMC2654324

[27] Sitzer M, Muller W, Siebler M, Hort W, Kniemeyer HW, Jancke L, et al Plaque ulceration and lumen thrombus are the main sources of cerebral microemboli in high-grade internal carotid artery stenosis. Stroke 1995;26:1231–3. 10.1161/01.str.26.7.1231 7604420

[28] Ferguson GG, Eliasziw M, Barr HW, Clagett GP, Barnes RW, Wallace MC, et al The North American Symptomatic Carotid Endarterectomy Trial: surgical results in 1415 patients. Stroke 1999;30:1751–8. 10.1161/01.str.30.9.1751 10471419

[29] Eliasziw M, Streifler JY, Fox AJ, Hachinski VC, Ferguson GG, Barnett HJ. Significance of plaque ulceration in symptomatic patients with high-grade carotid stenosis. North American Symptomatic Carotid Endarterectomy Trial. Stroke 1994;25:304–8. 10.1161/01.str.25.2.304 8303736

[30] Homburg PJ, Rozie S, van Gils MJ, van den Bouwhuijsen QJ, Niessen WJ, Dippel DW, et al Association between carotid artery plaque ulceration and plaque composition evaluated with multidetector CT angiography. Stroke 2011;42:367–72. 10.1161/STROKEAHA.110.597369 21183745

[31] Xu D, Hippe DS, Underhill HR, Oikawa-Wakayama M, Dong L, Yamada K, et al Prediction of high-risk plaque development and plaque progression with the carotid atherosclerosis score. JACC Cardiovasc Imaging 2014;7:366–73. 10.1016/j.jcmg.2013.09.022 24631510 PMC4046843

[32] Mono ML, Karameshev A, Slotboom J, Remonda L, Galimanis A, Jung S, et al Plaque characteristics of asymptomatic carotid stenosis and risk of stroke. Cerebrovasc Dis 2012;34:343–50. 10.1159/000343227 23154753

[33] Gupta A, Baradaran H, Schweitzer AD, Kamel H, Pandya A, Delgado D, et al Carotid plaque MRI and stroke risk: a systematic review and meta-analysis. Stroke 2013;44:3071–7. 10.1161/STROKEAHA.113.002551 23988640

[34] Coutinho JM, Derkatch S, Potvin AR, Tomlinson G, Kiehl TR, Silver FL, et al Nonstenotic carotid plaque on CT angiography in patients with cryptogenic stroke. Neurology 2016;87:665–72. 10.1212/WNL.0000000000002978 27412144 PMC4999163

[35] Zhao X, Hippe DS, Li R, Canton GM, Sui B, Song Y, et al Prevalence and characteristics of carotid artery high-risk atherosclerotic plaques in Chinese patients with cerebrovascular symptoms: a Chinese atherosclerosis risk evaluation II study. J Am Heart Assoc 2017;6:e005831. 10.1161/JAHA.117.005831 28862936 PMC5586432

[36] Yang J, Pan X, Zhang B, Yan Y, Huang Y, Woolf AK, et al Superficial and multiple calcifications and ulceration associate with intraplaque hemorrhage in the carotid atherosclerotic plaque. Eur Radiol 2018;28:4968–77. 10.1007/s00330-018-5535-7 29876705 PMC6223859

[37] Benson JC, Nardi V, Madhavan AA, Bois MC, Saba L, Savastano L, et al Reassessing the carotid artery plaque “rim sign” on CTA: a new analysis with histopathologic confirmation. AJNR Am J Neuroradiol 2022;43:429–34. 10.3174/ajnr.A7443 35210276 PMC8910788

[38] Eisenmenger LB, Aldred BW, Kim SE, Stoddard GJ, de Havenon A, Treiman GS, et al Prediction of carotid intraplaque hemorrhage using adventitial calcification and plaque thickness on CTA. AJNR Am J Neuroradiol 2016;37:1496–503. 10.3174/ajnr.A4765 27102316 PMC7960279

[39] Saba L, Chen H, Cau R, Rubeis GD, Zhu G, Pisu F, et al Impact analysis of different CT configurations of carotid artery plaque calcifications on cerebrovascular events. AJNR Am J Neuroradiol 2022;43:272–9. 10.3174/ajnr.A7401 35121588 PMC8985662

[40] Saba L, Lai ML, Montisci R, Tamponi E, Sanfilippo R, Faa G, et al Association between carotid plaque enhancement shown by multidetector CT angiography and histologically validated microvessel density. Eur Radiol 2012;22:2237–45. 10.1007/s00330-012-2467-5 22572988

[41] Song Y, Dang Y, Wang J, Cai H, Feng J, Zhang H, et al Carotid intraplaque neovascularization predicts ischemic stroke recurrence in patients with carotid atherosclerosis. Gerontology 2021;67:144–51. 10.1159/000511360 33582668

[42] Qiao Y, Etesami M, Astor BC, Zeiler SR, Trout HH III, Wasserman BA. Carotid plaque neovascularization and hemorrhage detected by MR imaging are associated with recent cerebrovascular ischemic events. AJNR Am J Neuroradiol 2012;33:755–60. 10.3174/ajnr.A2863 22194363 PMC3979429

[43] Romero JM, Babiarz LS, Forero NP, Murphy EK, Schaefer PW, Gonzalez RG, et al Arterial wall enhancement overlying carotid plaque on CT angiography correlates with symptoms in patients with high grade stenosis. Stroke 2009;40:1894–6. 10.1161/STROKEAHA.108.529008 19182087

[44] Poredos P, Gregoric ID, Jezovnik MK. Inflammation of carotid plaques and risk of cerebrovascular events. Ann Transl Med 2020;8:1281. 10.21037/atm-2020-cass-15 33178813 PMC7607075

[45] Saba L, Cau R, Murgia A, Nicolaides AN, Wintermark M, Castillo M, et al Carotid plaque-RADS: a novel stroke risk classification system. JACC Cardiovasc Imaging 2024;17:62–75. 10.1016/j.jcmg.2023.09.005 37823860

[46] Kamel H, Merkler AE, Iadecola C, Gupta A, Navi BB. Tailoring the approach to embolic stroke of undetermined source: a review. JAMA Neurol 2019;76:855–61. 10.1001/jamaneurol.2019.0591 30958521 PMC8078183

[47] Ntaios G, Papavasileiou V, Sagris D, Makaritsis K, Vemmos K, Steiner T, et al Closure of patent foramen ovale versus medical therapy in patients with cryptogenic stroke or transient ischemic attack. Updated systematic review and meta-analysis. Stroke 2018;49:412–8. 10.1161/strokeaha.117.020030 29335335

[48] Kent DM, Saver JL, Kasner SE, Nelson J, Carroll JD, Chatellier G, et al Heterogeneity of treatment effects in an analysis of pooled individual patient data from randomized trials of device closure of patent foramen ovale after stroke. JAMA 2021;326:2277–86. 10.1001/jama.2021.20956 34905030 PMC8672231

[49] Pristipino C, Sievert H, D'Ascenzo F, Mas JL, Meier B, Scacciatella P, et al European position paper on the management of patients with patent foramen ovale. General approach and left circulation thromboembolism. EuroIntervention 2019;14:1389–402. 10.4244/EIJ-D-18-00622 30141306

[50] Elgendy AY, Saver JL, Amin Z, Boudoulas KD, Carroll JD, Elgendy IY, et al Proposal for updated nomenclature and classification of potential causative mechanism in patent foramen ovale-associated stroke. JAMA Neurol 2020;77:878–86. 10.1001/jamaneurol.2020.0458 32282016

[51] Turc G, Lee JY, Brochet E, Kim JS, Song JK, Mas JL, et al Atrial septal aneurysm, shunt size, and recurrent stroke risk in patients with patent foramen ovale. J Am Coll Cardiol 2020;75:2312–20. 10.1016/j.jacc.2020.02.068 32381162

[52] Leung DY, Davidson PM, Cranney GB, Walsh WF. Thromboembolic risks of left atrial thrombus detected by transesophageal echocardiogram. Am J Cardiol 1997;79:626–9. 10.1016/s0002-9149(96)00828-4 9068521

[53] Lurie A, Wang J, Hinnegan KJ, McIntyre WF, Belley-Côté EP, Amit G, et al Prevalence of left atrial thrombus in anticoagulated patients with atrial fibrillation. J Am Coll Cardiol 2021;77:2875–86. doi: doi:10.1016/j.jacc.2021.04.036 34112315

[54] Noubiap JJ, Agbaedeng TA, Ndoadoumgue AL, Nyaga UF, Kengne AP. Atrial thrombus detection on transoesophageal echocardiography in patients with atrial fibrillation undergoing cardioversion or catheter ablation: a pooled analysis of rates and predictors. J Cardiovasc Electrophysiol 2021;32:2179–88. 10.1111/jce.15082 33969568

[55] Omran H, Rang B, Schmidt H, Illien S, Schimpf R, Maccarter D, et al Incidence of left atrial thrombi in patients in sinus rhythm and with a recent neurologic deficit. Am Heart J 2000;140:658–62. 10.1067/mhj.2000.109213 11011342

[56] Mac Grory B, Chang A, Atalay MK, Yaghi S. Left atrial appendage thrombus and embolic stroke. Stroke 2018;49:e286–9. 10.1161/STROKEAHA.118.022674 30355001 PMC6209112

[57] Fatkin D, Kelly RP, Feneley MP. Relations between left atrial appendage blood flow velocity, spontaneous echocardiographic contrast and thromboembolic risk in vivo. J Am Coll Cardiol 1994;23:961–9. 10.1016/0735-1097(94)90644-0 8106703

[58] Talner CN . Report of the New England Regional Infant Cardiac Program, by Donald C. Fyler, MD, pediatrics, 1980; 65(suppl):375–461. Pediatrics 1998;102:258–9. 9651450

[59] Hart RG, Pearce LA, Aguilar MI. Meta-analysis: antithrombotic therapy to prevent stroke in patients who have nonvalvular atrial fibrillation. Ann Intern Med 2007;146:857–67. 10.7326/0003-4819-146-12-200706190-00007 17577005

[60] Sposato LA, Cipriano LE, Saposnik G, Ruiz Vargas E, Riccio PM, Hachinski V. Diagnosis of atrial fibrillation after stroke and transient ischaemic attack: a systematic review and meta-analysis. Lancet Neurol 2015;14:377–87. 10.1016/s1474-4422(15)70027-x 25748102

[61] Schnabel RB, Haeusler KG, Healey JS, Freedman B, Boriani G, Brachmann J, et al Searching for atrial fibrillation poststroke: a white paper of the AF-SCREEN international collaboration. Circulation 2019;140:1834–50. 10.1161/circulationaha.119.040267 31765261

[62] Healey JS, Lopes RD, Granger CB, Alings M, Rivard L, McIntyre WF, et al Apixaban for stroke prevention in subclinical atrial fibrillation. N Engl J Med 2024;390:107–17. 10.1056/NEJMoa2310234 37952132

[63] Kirchhof P, Toennis T, Goette A, Camm AJ, Diener HC, Becher N, et al Anticoagulation with edoxaban in patients with atrial high-rate episodes. N Engl J Med 2023;389:1167–79. 10.1056/NEJMoa2303062 37622677

[64] Van Gelder IC, Healey JS, Crijns H, Wang J, Hohnloser SH, Gold MR, et al Duration of device-detected subclinical atrial fibrillation and occurrence of stroke in ASSERT. Eur Heart J 2017;38:1339–44. 10.1093/eurheartj/ehx042 28329139

[65] Singer DE, Ziegler PD, Koehler JL, Sarkar S, Passman RS. Temporal association between episodes of atrial fibrillation and risk of ischemic stroke. JAMA Cardiol 2021;6:1364–9. 10.1001/jamacardio.2021.3702 34586356 PMC8482300

[66] Singer AJ, Quinn A, Dasgupta N, Thode HC Jr. Management and outcomes of bleeding events in patients in the emergency department taking warfarin or a non-vitamin K antagonist oral anticoagulant. J Emerg Med 2017;52:1–7 e1. 10.1016/j.jemermed.2016.09.028 27793500

[67] McIntyre WF, Benz AP, Becher N, Healey JS, Granger CB, Rivard L, et al Direct oral anticoagulants for stroke prevention in patients with device-detected atrial fibrillation: a study-level meta-analysis of the NOAH-AFNET 6 and ARTESiA trials. Circulation 2023. 10.1161/CIRCULATIONAHA.123.067512 37952187

[68] Becher N, Toennis T, Bertaglia E, Blomström-Lundqvist C, Brandes A, Cabanelas N, et al Anticoagulation with edoxaban in patients with long atrial high-rate episodes ≥24 h. Eur Heart J 2023;45:837–849. 10.1093/eurheartj/ehad771. PMC10919916 37956458

[69] Perlepe K, Sirimarco G, Strambo D, Eskandari A, Karagkiozi E, Vemmou A, et al Left atrial diameter thresholds and new incident atrial fibrillation in embolic stroke of undetermined source. Eur J Intern Med 2020;75:30–4. 10.1016/j.ejim.2020.01.002 31952983

[70] Di Tullio MR, Sacco RL, Sciacca RR, Homma S. Left atrial size and the risk of ischemic stroke in an ethnically mixed population. Stroke 1999;30:2019–24. 10.1161/01.str.30.10.2019 10512901

[71] Yaghi S, Moon YP, Mora-McLaughlin C, Willey JZ, Cheung K, Di Tullio MR, et al Left atrial enlargement and stroke recurrence: the Northern Manhattan Stroke Study. Stroke 2015;46:1488–93. 10.1161/STROKEAHA.115.008711 25908460 PMC4442058

[72] Kamel H, O'Neal WT, Okin PM, Loehr LR, Alonso A, Soliman EZ. Electrocardiographic left atrial abnormality and stroke subtype in the atherosclerosis risk in communities study. Ann Neurol 2015;78:670–8. 10.1002/ana.24482 26179566 PMC4624007

[73] Kamel H, Okin PM, Elkind MS, Iadecola C. Atrial fibrillation and mechanisms of stroke: time for a new model. Stroke 2016;47:895–900. 10.1161/STROKEAHA.115.012004 26786114 PMC4766055

[74] Healey JS, Gladstone DJ, Swaminathan B, Eckstein J, Mundl H, Epstein AE, et al Recurrent stroke with rivaroxaban compared with aspirin according to predictors of atrial fibrillation: secondary analysis of the NAVIGATE ESUS randomized clinical trial. JAMA Neurol 2019;76:764–73. 10.1001/jamaneurol.2019.0617. 30958508 PMC6583060

[75] Kamel H, Longstreth WT Jr, Tirschwell DL, Kronmal RA, Marshall RS, Broderick JP, et al; ARCADIA Investigators. Apixaban to prevent recurrence after cryptogenic stroke in patients with atrial cardiopathy: the ARCADIA randomized clinical trial. JAMA 2024;331:573–581. 10.1001/jama.2023.27188 38324415 PMC10851142

[76] Yang M, Kondo T, Butt JH, Abraham WT, Anand IS, Desai AS, et al Stroke in patients with heart failure and reduced or preserved ejection fraction. Eur Heart J 2023;44:2998–3013. 10.1093/eurheartj/ehad338 37358785 PMC10424882

[77] Cohen A, Donal E, Delgado V, Pepi M, Tsang T, Gerber B, et al EACVI recommendations on cardiovascular imaging for the detection of embolic sources: endorsed by the Canadian Society of Echocardiography. Eur Heart J Cardiovasc Imaging 2021;22:e24–57. 10.1093/ehjci/jeab008 33709114

[78] Homma S, Thompson JL, Pullicino PM, Levin B, Freudenberger RS, Teerlink JR, et al Warfarin and aspirin in patients with heart failure and sinus rhythm. N Engl J Med 2012;366:1859–69. 10.1056/NEJMoa1202299 22551105 PMC3723382

[79] Li W, Seo J, Kokkinidis DG, Palaiodimos L, Nagraj S, Korompoki E, et al Efficacy and safety of vitamin-K antagonists and direct oral anticoagulants for stroke prevention in patients with heart failure and sinus rhythm: an updated systematic review and meta-analysis of randomized clinical trials. Int J Stroke 2023;18:392–9. 10.1177/17474930221109149 35689348

[80] El-Am EA, Dispenzieri A, Grogan M, Nkomo VT. Reply: cardiac amyloidosis, atrial arrhythmias, thrombus, and stroke: a vexing problem. J Am Coll Cardiol 2019;73:2911–3. 10.1016/j.jacc.2019.04.005 31171105

[81] Habib G, Charron P, Eicher JC, Giorgi R, Donal E, Laperche T, et al Isolated left ventricular non-compaction in adults: clinical and echocardiographic features in 105 patients. Results from a French registry. Eur J Heart Fail 2011;13:177–85. 10.1093/eurjhf/hfq225 21193437

[82] Levine GN, McEvoy JW, Fang JC, Ibeh C, McCarthy CP, Misra A, et al Management of patients at risk for and with left ventricular thrombus: a scientific statement from the American Heart Association. Circulation 2022;146:e205–23. 10.1161/CIR.0000000000001092 36106537

[83] Towbin JA, McKenna WJ, Abrams DJ, Ackerman MJ, Calkins H, Darrieux FCC, et al 2019 HRS expert consensus statement on evaluation, risk stratification, and management of arrhythmogenic cardiomyopathy: executive summary. Heart Rhythm 2019;16:e373–407. 10.1016/j.hrthm.2019.09.019 31676023

[84] Chen L, Yan S, He Y, Zhong W, Gong X, Lou M, et al Prediction of acute myocardial infarction in Asian patients with acute ischemic stroke: the CTRAN score. JACC Asia 2022;2:845–52. 10.1016/j.jacasi.2022.08.008 36713755 PMC9876956

[85] Boivin-Proulx LA, Ieroncig F, Demers SP, Nozza A, Soltani M, Ghersi I, et al Contemporary incidence and predictors of left ventricular thrombus in patients with anterior acute myocardial infarction. Clin Res Cardiol 2023;112:558–65. 10.1007/s00392-023-02158-8 36651998

[86] Thuny F, Di Salvo G, Belliard O, Avierinos JF, Pergola V, Rosenberg V, et al Risk of embolism and death in infective endocarditis: prognostic value of echocardiography: a prospective multicenter study. Circulation 2005;112:69–75. 10.1161/CIRCULATIONAHA.104.493155 15983252

[87] Arregle F, Martel H, Philip M, Gouriet F, Casalta JP, Riberi A, et al Infective endocarditis with neurological complications: delaying cardiac surgery is associated with worse outcome. Arch Cardiovasc Dis 2021;114:527–36. 10.1016/j.acvd.2021.01.004 33935000

[88] Quintero-Martinez JA, Hindy JR, El Zein S, Michelena HI, Nkomo VT, DeSimone DC, et al Contemporary demographics, diagnostics and outcomes in non-bacterial thrombotic endocarditis. Heart 2022;108:heartjnl-2022-320970. 10.1136/heartjnl-2022-320970. 35534050

[89] Breithardt G, Baumgartner H, Berkowitz SD, Hellkamp AS, Piccini JP, Stevens SR, et al Clinical characteristics and outcomes with rivaroxaban vs. warfarin in patients with non-valvular atrial fibrillation but underlying native mitral and aortic valve disease participating in the ROCKET AF trial. Eur Heart J 2014;35:3377–85. 10.1093/eurheartj/ehu305 25148838 PMC4265383

[90] Steiner DK, Sogaard P, Jensen M, Bjerregaard Larsen T, Lip GYH, Nielsen PB. Risk of stroke or systemic embolism in patients with degenerative mitral stenosis with or without atrial fibrillation: a cohort study. Int J Cardiol Heart Vasc 2022;43:101126. 10.1016/j.ijcha.2022.101126 36237964 PMC9550603

[91] Okura H, Nakada Y, Nogi M, Ishihara S, Okamura A, Okayama S, et al Prevalence of mitral annular calcification and its association with mitral valvular disease. Echocardiography 2021;38:1907–12. 10.1111/echo.15236 34719060

[92] Suhai FI, Varga A, Szilveszter B, Nagy-Vecsey M, Apor A, Nagy AI, et al Predictors and neurological consequences of periprocedural cerebrovascular events following transcatheter aortic valve implantation with self-expanding valves. Front Cardiovasc Med 2022;9:951943. 10.3389/fcvm.2022.951943 36277778 PMC9581280

[93] Ishizuka K, Hoshino T, Ashihara K, Mruyama K, Toi S, Mizuno S, et al Associations of mitral and aortic valve calcifications with complex aortic atheroma in patients with embolic stroke of undetermined source. J Stroke Cerebrovasc Dis 2018;27:697–702. 10.1016/j.jstrokecerebrovasdis.2017.09.057 29174290

[94] Leitman M, Tyomkin V, Peleg E, Shmueli R, Krakover R, Vered Z. Clinical significance and prevalence of valvular strands during routine echo examinations. Eur Heart J Cardiovasc Imaging 2014;15:1226–30. 10.1093/ehjci/jeu110 24939951

[95] Salehi Omran S, Chaker S, Lerario MP, Merkler AE, Navi BB, Kamel H. Relationship between Lambl's excrescences and embolic strokes of undetermined source. Eur Stroke J 2020;5:169–73. 10.1177/2396987319901201 32637650 PMC7313363

[96] Navi BB, Iadecola C. Ischemic stroke in cancer patients: a review of an underappreciated pathology. Ann Neurol 2018;83:873–83. 10.1002/ana.25227 29633334 PMC6021225

[97] Navi BB, Singer S, Merkler AE, Cheng NT, Stone JB, Kamel H, et al Recurrent thromboembolic events after ischemic stroke in patients with cancer. Neurology 2014;83:26–33. 10.1212/WNL.0000000000000539 24850486 PMC4114176

[98] Navi BB, Reiner AS, Kamel H, Iadecola C, Okin PM, Tagawa ST, et al Arterial thromboembolic events preceding the diagnosis of cancer in older persons. Blood 2019;133:781–9. 10.1182/blood-2018-06-860874 30578253 PMC6384185

[99] Selvik HA, Bjerkreim AT, Thomassen L, Waje-Andreassen U, Naess H, Kvistad CE. When to screen ischaemic stroke patients for cancer. Cerebrovasc Dis 2018;45:42–7. 10.1159/000484668 29402826

[100] Gon Y, Sakaguchi M, Takasugi J, Kawano T, Kanki H, Watanabe A, et al Plasma D-dimer levels and ischaemic lesions in multiple vascular regions can predict occult cancer in patients with cryptogenic stroke. Eur J Neurol 2017;24:503–8. 10.1111/ene.13234 28026909

[101] Cocho D, Gendre J, Boltes A, Espinosa J, Ricciardi AC, Pons J, et al Predictors of occult cancer in acute ischemic stroke patients. J Stroke Cerebrovasc Dis 2015;24:1324–8. 10.1016/j.jstrokecerebrovasdis.2015.02.006 25881772

[102] Beyeler M, Birner B, Branca M, Meinel T, Vynckier J, Buffle E, et al Development of a score for prediction of occult malignancy in stroke patients (occult-5 score). J Stroke Cerebrovasc Dis 2022;31:106609. 10.1016/j.jstrokecerebrovasdis.2022.106609 35753093

[103] Navi BB, Reiner AS, Kamel H, Iadecola C, Elkind MS, Panageas KS, et al Association between incident cancer and subsequent stroke. Ann Neurol 2015;77:291–300. 10.1002/ana.24325 25472885 PMC4315703

[104] Otite FO, Somani S, Aneni E, Akano E, Patel SD, Anikpezie N, et al Trends in age and sex-specific prevalence of cancer and cancer subtypes in acute ischemic stroke from 2007–2019. J Stroke Cerebrovasc Dis 2022;31:106818. 10.1016/j.jstrokecerebrovasdis.2022.106818 36323171

[105] Navi BB, Kasner SE, Elkind MSV, Cushman M, Bang OY, DeAngelis LM. Cancer and embolic stroke of undetermined source. Stroke 2021;52:1121–30. 10.1161/STROKEAHA.120.032002 33504187 PMC7902455

[106] Navi BB, Sherman CP, Genova R, Mathias R, Lansdale KN, LeMoss NM, et al Mechanisms of ischemic stroke in patients with cancer: a prospective study. Ann Neurol 2021;90:159–69. 10.1002/ana.26129 34029423 PMC9297305

[107] Graus F, Rogers LR, Posner JB. Cerebrovascular complications in patients with cancer. Medicine (Baltimore) 1985;64:16–35. 10.1097/00005792-198501000-00002 3965856

[108] Hurrell H, Roberts-Thomson R, Prendergast BD. Non-infective endocarditis. Heart 2020;106:1023–9. 10.1136/heartjnl-2019-315204 32376608

[109] Merkler AE, Navi BB, Singer S, Cheng NT, Stone JB, Kamel H, et al Diagnostic yield of echocardiography in cancer patients with ischemic stroke. J Neurooncol 2015;123:115–21. 10.1007/s11060-015-1768-3 25851114 PMC4441574

[110] Park H, Kim J, Ha J, Hwang IG, Song TJ, Yoo J, et al Histological features of intracranial thrombi in stroke patients with cancer. Ann Neurol 2019;86:143–9. 10.1002/ana.25495 31025392

[111] Taylor FB Jr, Toh CH, Hoots WK, Wada H, Levi M; Scientific Subcommittee on Disseminated Intravascular Coagulation (DIC) of the International Society on Thrombosis and Haemostasis (ISTH). Towards definition, clinical and laboratory criteria, and a scoring system for disseminated intravascular coagulation. Thromb Haemost 2001;86:1327–30. 11816725

[112] Bang OY, Chung JW, Cho YH, Oh MJ, Seo WK, Kim GM, et al Circulating DNAs, a marker of neutrophil extracellular traposis and cancer-related stroke: the OASIS-cancer study. Stroke 2019;50:2944–7. 10.1161/STROKEAHA.119.026373 31394991

[113] Gon Y, Okazaki S, Terasaki Y, Sasaki T, Yoshimine T, Sakaguchi M, et al Characteristics of cryptogenic stroke in cancer patients. Ann Clin Transl Neurol 2016;3:280–7. 10.1002/acn3.291 27081658 PMC4818743

[114] Schwarzbach CJ, Schaefer A, Ebert A, Held V, Bolognese M, Kablau M, et al Stroke and cancer: the importance of cancer-associated hypercoagulation as a possible stroke etiology. Stroke 2012;43:3029–34. 10.1161/STROKEAHA.112.658625 22996958

[115] Nam KW, Kim CK, Kim TJ, An SJ, Demchuk AM, Kim Y, et al D-dimer as a predictor of early neurologic deterioration in cryptogenic stroke with active cancer. Eur J Neurol 2017;24:205–11. 10.1111/ene.13184 27766716

[116] Finelli PF, Nouh A. Three-territory DWI acute infarcts: diagnostic value in cancer-associated hypercoagulation stroke (Trousseau syndrome). AJNR Am J Neuroradiol 2016;37:2033–6. 10.3174/ajnr.A4846 27365322 PMC7963789

[117] Navi BB, Zhang C, Sherman CP, Genova R, LeMoss NM, Kamel H, et al Ischemic stroke with cancer: hematologic and embolic biomarkers and clinical outcomes. J Thromb Haemost 2022;20:2046–57. 10.1111/jth.15779 35652416 PMC9378694

[118] Seok JM, Kim SG, Kim JW, Chung CS, Kim GM, Lee KH, et al Coagulopathy and embolic signal in cancer patients with ischemic stroke. Ann Neurol 2010;68:213–9. 10.1002/ana.22050 20695014

[119] Rioux B, Touma L, Nehme A, Gore G, Keezer MR, Gioia LC. Frequency and predictors of occult cancer in ischemic stroke: a systematic review and meta-analysis. Int J Stroke 2021;16:12–9. 10.1177/1747493020971104 33197367

[120] Hasegawa Y, Setoguchi T, Sakaida T, Iuchi T. Utility of a scoring system for differentiating cancer-associated stroke from cryptogenic stroke in patients with cancer. Neurol Sci 2020;41:1245–50. 10.1007/s10072-019-04231-5 31912335

[121] Nezu T, Hosomi N, Naito H, Aoki S, Torii T, Kurashige T, et al Clinical characteristics and tumor markers in ischemic stroke patients with active cancer. Intern Emerg Med 2022;17:735–41. 10.1007/s11739-021-02862-1 34596824

[122] Abdelsalam M, Abu-Hegazy M, El-Hadaad HA, Wahba H, Egila H, Esmael A. Pathophysiology, mechanism, and outcome of ischemic stroke in cancer patients. J Stroke Cerebrovasc Dis 2020;29:105299. 10.1016/j.jstrokecerebrovasdis.2020.105299 32951960

[123] Navi BB, Kawaguchi K, Hriljac I, Lavi E, DeAngelis LM, Jamieson DG. Multifocal stroke from tumor emboli. Arch Neurol 2009;66:1174–5. 10.1001/archneurol.2009.172 19752313

[124] Kitano T, Sasaki T, Gon Y, Todo K, Okazaki S, Kitamura T, et al The effect of chemotherapy on stroke risk in cancer patients. Thromb Haemost 2020;120:714–23. 10.1055/s-0040-1708484 32289866

[125] Parikh NS, Burch JE, Kamel H, DeAngelis LM, Navi BB. Recurrent thromboembolic events after ischemic stroke in patients with primary brain tumors. J Stroke Cerebrovasc Dis 2017;26:2396–403. 10.1016/j.jstrokecerebrovasdis.2017.05.031 28647417 PMC5600665

[126] Ntaios G, Omran SS. Diagnostic challenges and uncertainties of embolic strokes of undetermined source in young adults. JAMA Neurol 2022;79:444–7. 10.1001/jamaneurol.2022.0058 35285865

[127] Ntaios G, Perlepe K, Lambrou D, Sirimarco G, Strambo D, Eskandari A, et al Identification of patients with embolic stroke of undetermined source and low risk of new incident atrial fibrillation: the AF-ESUS score. Int J Stroke 2021;16:29–38. 10.1177/1747493020925281 32423317

[128] Kitsiou A, Sagris D, Schäbitz WR, Ntaios G. Validation of the AF-ESUS score to identify patients with embolic stroke of undetermined source and low risk of device-detected atrial fibrillation. Eur J Intern Med 2021;89:135–6. 10.1016/j.ejim.2021.04.003 33952425

[129] Papanagiotou P, Ntaios G. Endovascular thrombectomy in acute ischemic stroke. Circ Cardiovasc Interv 2018;11:e005362. 10.1161/circinterventions.117.005362 29311286

[130] Hart RG, Sharma M, Mundl H, Kasner SE, Bangdiwala SI, Berkowitz SD, et al Rivaroxaban for stroke prevention after embolic stroke of undetermined source. N Engl J Med 2018;378:2191–201. 10.1056/NEJMoa1802686 29766772

[131] Diener HC, Sacco RL, Easton JD, Granger CB, Bernstein RA, Uchiyama S, et al Dabigatran for prevention of stroke after embolic stroke of undetermined source. N Engl J Med 2019;380:1906–17. 10.1056/NEJMoa1813959 31091372

[132] Poli S, Meissner C, Baezner HJ, Kraft A, Hillenbrand F, Hobohm C, et al Apixaban for treatment of embolic stroke of undetermined source (ATTICUS) randomized trial—update of patient characteristics and study timeline after interim analysis. Eur Heart J 2021;42:ehab724.2070. 10.1093/eurheartj/ehab724.2070

